# Filled and empty states of Zn-TPP films deposited on Fe(001)-*p*(1×1)O

**DOI:** 10.3762/bjnano.7.146

**Published:** 2016-10-27

**Authors:** Gianlorenzo Bussetti, Alberto Calloni, Rossella Yivlialin, Andrea Picone, Federico Bottegoni, Marco Finazzi

**Affiliations:** 1 Department of Physics, Politecnico di Milano, p.za Leonardo da Vinci 32, 20133 Milano, Italy

**Keywords:** inverse photoemission, metal-oxide film, OMBE, porphyrin

## Abstract

Zn-tetraphenylporphyrin (Zn-TPP) was deposited on a single layer of metal oxide, namely an Fe(001)-*p*(1×1)O surface. The filled and empty electronic states were measured by means of UV photoemission and inverse photoemission spectroscopy on a single monolayer and a 20 monolayer thick film. The ionization energy and the electron affinity of the organic film were deduced and the interface dipole was determined and compared with data available in the literature.

## Introduction

**Figure 1:**
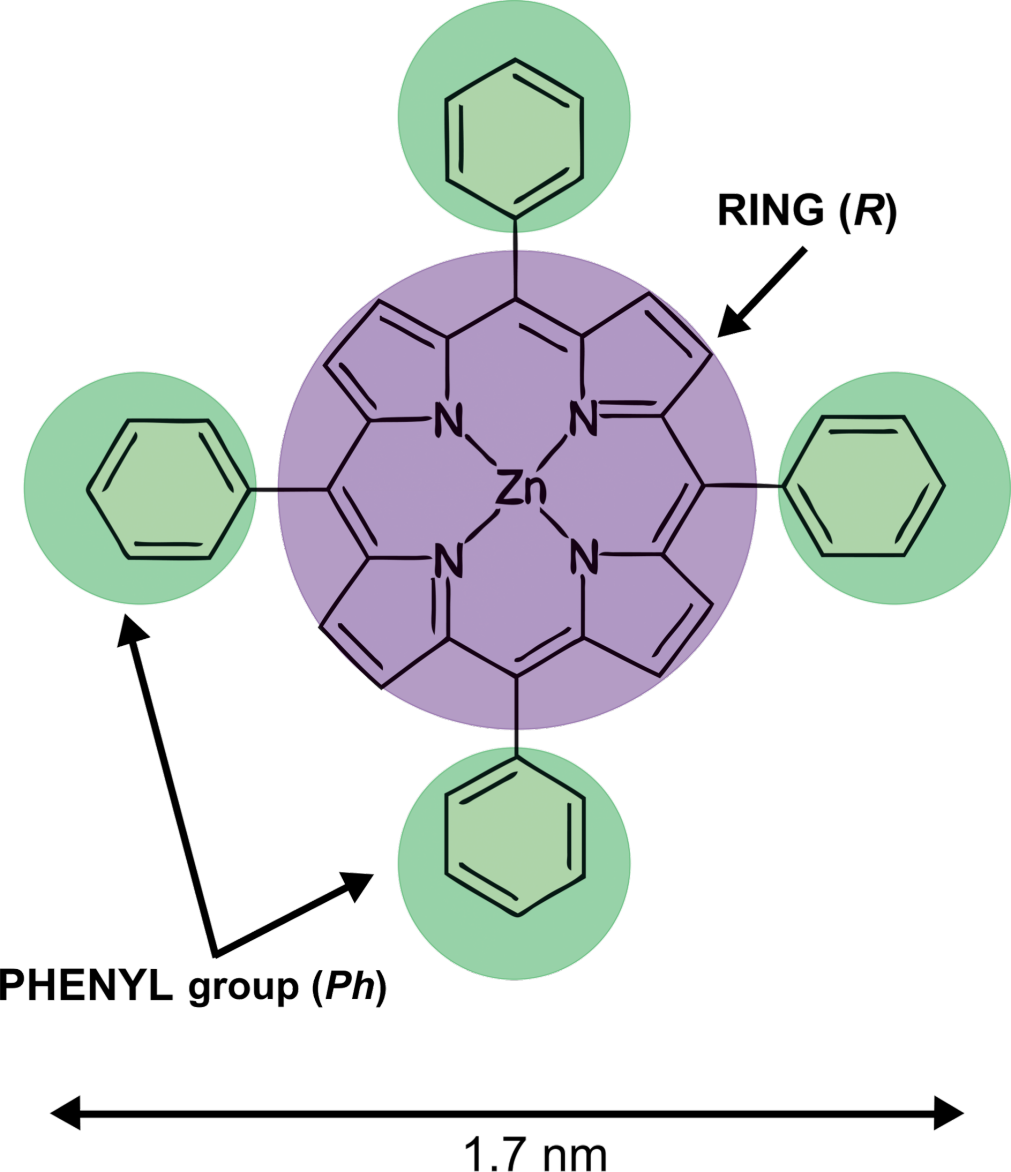
The structure of the Zn-tetraphenylporphyrin molecule. The main inner cavity of the porphyrin (ring) as well as the four phenyl groups have been marked in the image.

Thin organic films can be realized by depositing single molecules on surfaces, which is the first step for the so-called bottom-up assembly of devices based on organic compounds. The molecule–surface interaction, however, can alter the electronic properties of the organic compound and/or the functionality of the electronic device. This effect is enhanced in molecules showing catalytic activity when the catalytic sites directly interact with the substrate [1]. A characteristic example is offered by metal-tetraphenylporphyrins (M-TPPs). These molecules have been studied in many research fields [2–6] because a specific change in their peripheral groups or inner metal ion can induce enormous variations in the porphyrin reactivity [1]. In particular, the metal atom is placed in the middle of the main cavity of the porphyrin, which has a planar structure, allowing the metal atom to interact from both sides of the molecule. The molecule–substrate interaction can be interpreted in terms of a bond between a special ligand (the surface) and the porphyrin (the so-called surface trans effect (STE)) [1,7]. In order to avoid this problem, porphyrin films are usually grown on passivated surfaces [1] or, conversely, thick (on the order of a few nanometers) films are exploited [8]. A possible alternative is the use of ultrathin metal oxide (MO) films [9]. Here, a single layer of oxygen atoms can decouple, or at least reduce, the interaction between the grown molecules and the buried metal substrate. The mechanisms involved during the film growth on the oxide layer are still under debate. In this respect, we have recently studied the growth of Zn-TPP (the molecular structure is reported in Figure 1), a well-characterized and studied porphyrin, on a prototypical ultrathin MO substrate, namely Fe(001)-*p*(1×1)O [10]. On this surface, oxygen atoms are placed between four metal atoms, slightly above the Fe(001) uppermost layer, making an ultrathin Fe monoxide layer. From our data we observe an increase of the porphyrin diffusivity on the MO layer [12]. This allows molecules to assemble in an ordered square super-lattice showing a (5 × 5) reconstruction, as observed by low-energy electron diffraction. An X-ray photoemission analysis proves that Zn-TPP molecules are deposited flat on the surface and the molecular skeleton is not significantly distorted, as observed when Zn-TPP is grown on other substrates for comparison.

In this paper, we investigate the electronic structure of a Zn-TPP film, studying both normally occupied and unoccupied molecular levels by using ultraviolet photoemission (UPS) and inverse photoemission spectroscopy (IPES), respectively. A comparison between filled and empty states can help to reveal the creation and the value of an interface dipole, which shifts the sample vacuum energy level with respect to the pristine Fe(001)-*p*(1×1)O substrate. The determination of such an interface dipole, its direction with respect to the sample surface, and its dependence on the porphyrin film thickness are important in view of possible applications in electronic device prototypes. In such devices, the band alignment between molecular levels and substrate bands plays a key role in the transport properties.

## Results and Discussion

From a technological point of view, the 1 monolayer (ML) thick sample is the most interesting and appealing, due to its ordered (5 × 5) reconstruction [10] that can be exploited (i) as a template for the deposition of other organic molecules or (ii) as a buffer layer in flat organic devices. On the other hand, a detailed analysis of the electronic properties of the porphyrin single layer requires a reference sample for comparison. Generally, as well as in this paper, a thick (typically 20 ML) porphyrin film is used for this purpose [10–11]. There, the substrate is almost completely covered by porphyrins. The spectra acquired on thick films can be considered representative of the electronic properties of a hypothetical isolated molecule, since molecule–molecule interactions are limited to weak van der Waals forces [11]. Consequently, changes in the energy position of the different spectroscopic features of the 1 ML film with respect to the reference layer are usually interpreted in terms of intensity strength of the molecule–substrate interaction.

**Figure 2:**
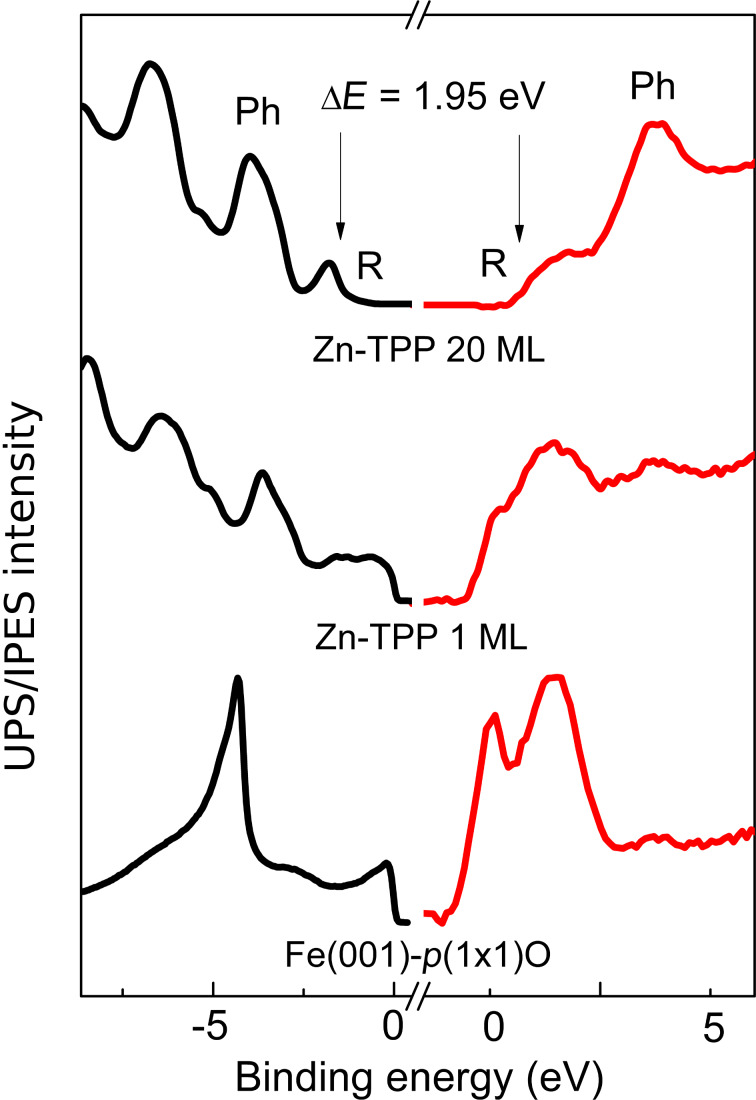
Filled (black lines) and empty (red lines) states acquired on freshly prepared Fe(001)-*p*(1×1)O for 1 ML and 20 ML thick Zn-TPP samples.

In Figure 2, we report the filled (black line) and empty (red line) states of the Fe(001)-*p*(1×1)O substrate. The filled states are characterized by an intense peak at about 4.5 eV, due to the O 2p states of the oxygen layer [12]. On the other hand, the empty states are dominated by two peaks, close to the Fermi energy level, which are distinctive structures well known for their spin-polarized character [13]. The small feature at about 4.0 eV is usually attributed to an image state resonance that demonstrates the very good quality of the surface preparation [14].

The 20 ML thick sample shows the main features of the Zn-TPP molecule, where the different peaks are visible in the filled states. The first structure, close to the Fermi energy, is related to the HOMO level of the main molecular ring (at 1.76 eV with respect to the Fermi level and labelled R in the figure), while the intense structures at 4.0 eV and 6.7 eV are linked to the phenyl groups (Ph) of the molecule [10]. In the empty states, we recognize two features, one at about 1.5 eV (onset at 0.75 eV), and the second at 3.7 eV. We ascribe them to R-LUMO and Ph-LUMO, respectively. These results, and the states assignments, are in close agreement with data reported on a comparable molecule, Zn-phthalocyanine (Zn-Pc) [14]. In the Zn-Pc film, a gap of about 1.94 eV [14] is measured between the onset of the *R*-HOMO peak and the onset of the R-LUMO feature, in very good agreement with our data (1.95 eV). As discussed in detail in [10], the porphyrin R-HOMO and Ph-HOMO are already visible in the 1 ML thick sample. The empty states of the ultrathin layer are affected however by the signal arising from the buried substrate. The IPES spectrum of the 1 ML thick sample is dominated by structures close to the Fermi energy, which are similar to the two peaks of the clean substrate. At 3.6 eV, a quite large feature appears. Considering the observed energy shift of the Zn-TPP peaks between 1 ML and 20 ML structures [10], we attribute this structure to the Ph-LUMO state.

**Figure 3:**
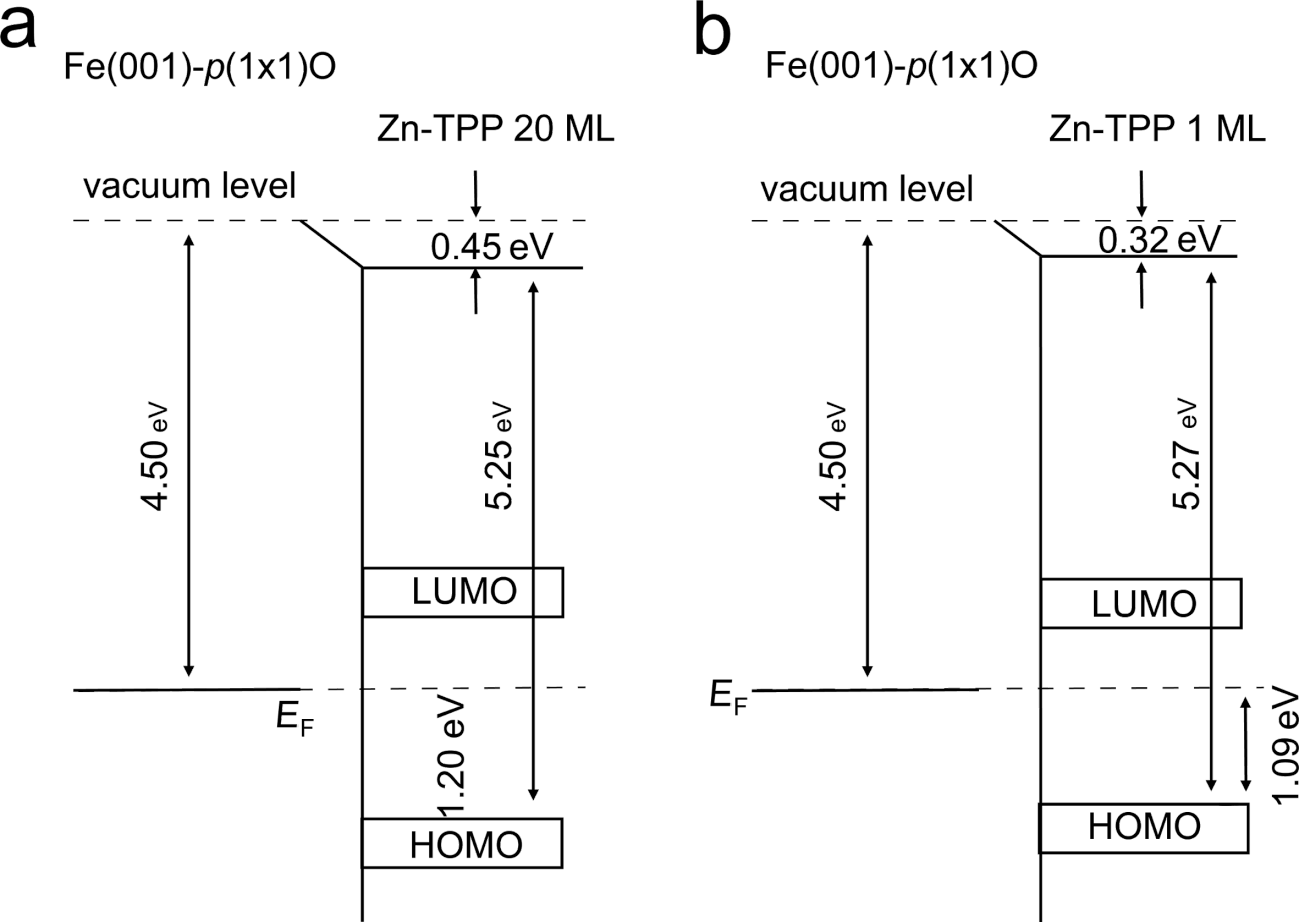
Energy of molecular levels near the interface between Fe(001)-*p*(1×1)O and the (a) 20 ML thick Zn-TPP film and (b) 1 ML thick Zn-TTP film. The interface dipole, work function of the bare substrate, and Zn-TPP ionization energy are indicated.

From the data acquired with UPS and IPES measurements, the ionization energy (the difference between the vacuum level, *E*_vac_, and the leading edge of the HOMO) and the electron affinity (the difference between *E*_vac_ and the LUMO) of the condensed organic film can be deduced. For this purpose, we have measured the sample work function from the energy position of the low-energy secondary electron cutoff edge that, compared with the work function (WF) of the pristine Fe(001)-*p*(1×1)O (4.50 eV [13]), allows the determination of the interface dipole, as reported in Figure 3.

The results obtained for the 20 ML film are in good agreement with those reported for Zn-Pc [14] deposited on a gold substrate. The measured ionization energy (5.25 eV, see Figure 3a) is directly comparable with the value obtained for Zn-Pc (5.28 eV) [14], confirming that Zn-TPP and Zn-Pc have a comparable electronic as well as chemical structure. Conversely, the interface dipole of the Zn-TPP film (0.45 eV, see Figure 3a) is about 300 meV smaller with respect to the Zn-Pc film (0.76 eV, as reported in [14]), suggesting a different (lower) molecule–substrate interaction. The sample belongs to the large organic/metal interface group at which the vacuum level alignment rule breaks down [15] and chemical bonds play a key role in tuning the barrier height [16]. With these interfaces, the sign of the dipole is deduced from the decreasing substrate work function and interpreted in terms of a (partial) electron transfer from the organic material to the Fe(001)-*p*(1×1)O surface [14]. In this picture, the direction of the dipole vector points from the substrate into the (positive) organic film. In Figure 3b, a similar analysis is reported for the 1 ML sample. In this case, an interface dipole of about 0.32 meV is found. This means that its value increases as a function of the deposited organic film thickness (0.32 meV at 1 ML vs 0.45 meV at 20 ML) in agreement with data reported in the literature [17]. The determination of the LUMO level is here more critical, because, as mentioned above, the IPES spectra are partially affected by the substrate photoemission signal. The LUMO energy position has been assessed after an analysis of the acquired spectra, the details of which are reported in Supporting Information File 1. As recently reported by the authors [10], the Zn-TPP sample undergoes a phase transition (from a (5 × 5) to a (√5 × √5) reconstruction) for film thicknesses larger than 1 ML. Generally speaking, a change in the molecular packing could influence the energy levels of the film. However, the band alignment at the molecule/substrate interface is mainly due to a charge transfer between the organic layers and the substrate and a consequent modification of the electron density, as reported in the literature [18]. The changes of the interface dipole, as a function of the film thickness, can thus give a first characterization to evaluate possible barriers that affect the transport properties of the junction. Finally, we summarize in the Table 1 the main energy positions of the R/Ph-HOMO and R/Ph-LUMO molecular levels as measured and/or deduced from our data.

**Table 1:** Binding energy position of both filled and empty molecular levels as a function of the porphyrin film thickness.

	1 ML thick sample	20 ML thick sample

R-HOMO	1.50 ± 0.01 eV	1.76 ± 0.01 eV
Ph-HOMO	3.80 ± 0.01 eV	4.00 ± 0.01 eV
R-LUMO	0.6 ± 0.3 eV	0.8 ± 0.3 eV
Ph-LUMO	3.6 ± 0.3 eV	3.7 ± 0.3 eV

## Conclusion

An organic Zn-TPP film was grown under UHV conditions in a special chamber devoted to the sublimation of molecules. The porphyrin films were deposited at RT on a freshly prepared Fe(001)-*p*(1×1)O substrate, whose topmost layer can be considered prototypical of the wide class of thin MO films. The ultrathin oxide layer is able to decouple the molecules from the buried iron substrate. The reduced molecule–substrate interaction allows preservation of the main electronic properties of the Zn-TPP porphyrins. This means that the HOMO and LUMO levels of the organic film are placed close to the characteristic energy values of the unperturbed molecule. In this paper, the filled and empty states of the organic film were studied and the formation of the interface dipole was analyzed. These results are interesting in view of applications of ultrathin Zn-TPP films in organic devices, where the alignment of the HOMO and LUMO levels of the molecule with the substrate bands play a crucial role in charge transport.

## Experimental

The experimental apparatus consists of a multichamber ultrahigh vacuum (UHV, base pressure in the 10^−8^ Pa range) system described elsewhere [19], coupled to a chamber devoted to organic molecular beam epitaxy (OMBE). The OMBE chamber was designed and built in collaboration with 5Pascal srl. (via Boccaccio 108, 20090 Trezzano sul Naviglio, Milano, Italy). The OMBE system is equipped with four Knudsen cells, whose crucibles are controlled within 0.5 °C. One of the cells is filled with Zn-TPP molecules provided by Sigma-Aldrich and purified in vacuum by several cycles of annealing at 150 °C and flashes at 310 °C, until the pressure in the OMBE chamber was stable in the low 10^−7^ Pa range. The molecule sublimation was achieved at a temperature of 300 °C and the molecular flux (0.5 ML/min, where 1 ML is 3.06 Å [11]) was measured by a quartz microbalance. The Fe(001) substrate was kept at room temperature during the porphyrin sublimation.

The Fe(001)-*p*(1×1)O fresh surface was prepared by exposing the clean Fe(001) surface to few Langmuir of molecular oxygen followed by annealing at 630 °C, as reported in the literature [20–21].

Ultraviolet photoemission spectroscopy (UPS) was performed by exciting electrons out of the sample at normal emission with a UV radiation (*h*ν = 21.2 eV) and detecting them by means of a 150 mm hemispherical analyzer (SPECS GmbH) [11], having an energy resolution of about 50 meV. A GaAs(001) photocathode, prepared according to standard procedures [22–23], was used for inverse photoemission spectroscopy (IPES), operating in the isochromatic mode, by detecting 9.6 eV photons with a band-pass detector [24–26]. The IPES energy resolution is about 700 meV. All the experiments reported here were achieved under negligible charging conditions during electron spectroscopy data acquisition. The position of the vacuum level was obtained by adding the photon energy to the low-energy secondary electron cutoff acquired with the sample at negative bias (−10 V).

## Supporting Information

File 1 Additional experimental information.
