# Supplementary material for: Filled and empty states of Zn-TPP films deposited on Fe(001)-p(1×1)O
Source: Beilstein J Nanotechnol. 2016 Oct 27;7:1527–31. doi: 10.3762/bjnano.7.146 (PMC5238660; doi:10.3762/bjnano.7.146)
Supplement: File 1 — Additional experimental information. [file Beilstein_J_Nanotechnol-07-1527-s001.pdf]

Supporting Information

for

Filled and empty states of Zn-TPP films deposited on Fe(001)-*p*(1×1)O

Gianlorenzo Bussetti*, Alberto Calloni, Rossella Yivlialin, Andrea Picone, Federico Bottegoni and Marco Finazzi

Address: Department of Physics, Politecnico di Milano, p.za Leonardo da Vinci 32, 20133 Milano, Italy

Email: Gianlorenzo Bussetti - gianlorenzo.bussetti@polimi.it

*Corresponding author

Additional experimental information

The determination of the *R*-LUMO level from the spectra acquired on the 1 ML-thick porphyrin film is complicated by the fact that the characteristic features of the bare substrate still affect the IPES line shape, as reported in Figure 1a.

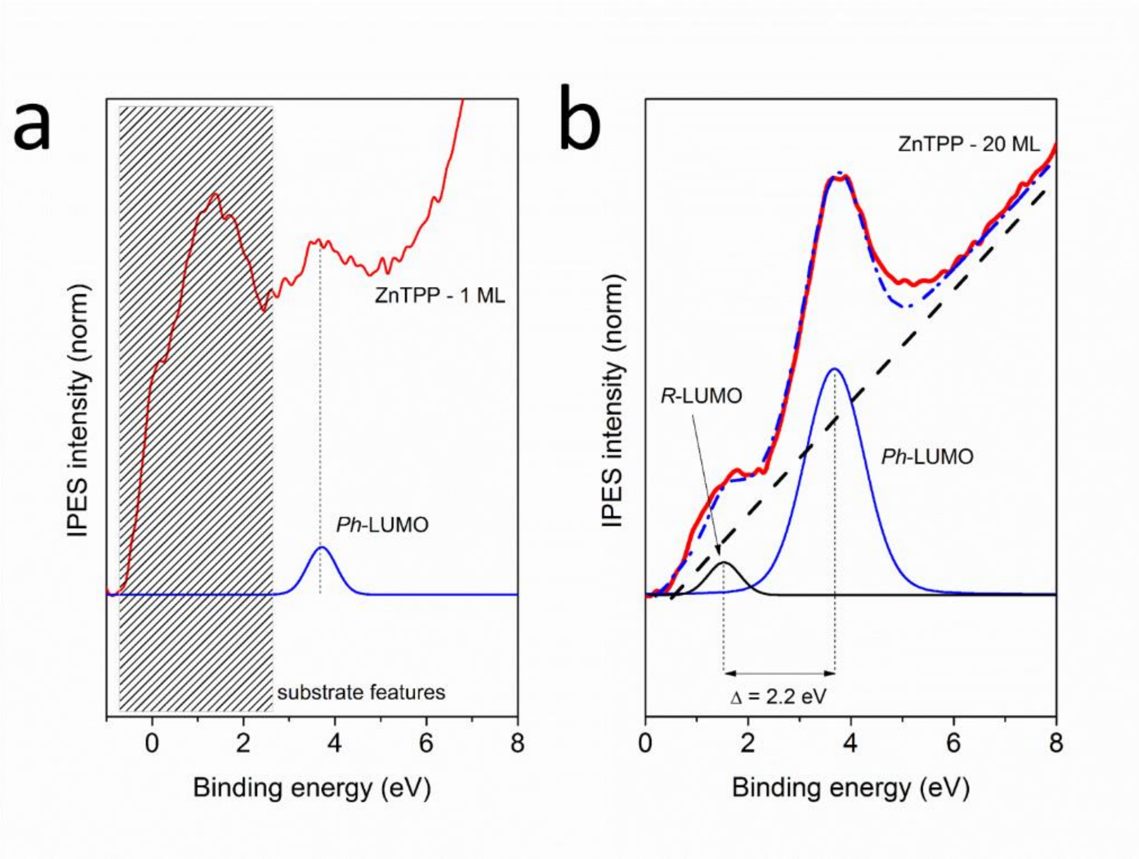

Figure 1: analysis of the IPES spectra acquired on (a) 1 ML-thick and (b) 20 ML-thick samples. In panel b, the linear background (black dash line) and the spectrum fit (blue dash-dot line) are reported.

The feature placed at 3.7 eV has been fitted by a Voigt curve. As reported in the main text, we identify this feature with the *Ph*-LUMO level. To evaluate the *R*-LUMO position, we have fitted the IPES 20 ML sample, where signals from the substrate do not affect the line shape, with two Voigt curves after a linear background subtraction (see Figure 1b). The energy position of both the *R*-

LUMO and the *Ph*-LUMO is easily identified and reported in Table 1 (see the text). The *Ph*-LUMO is slightly shifted towards higher binding energies (BE) with respect to the 1 ML sample. This shift is comparable to what already observed in the filled states of phenyl groups [S1]. Moreover, the authors have proven that the ZnTPP *R*-HOMO shifts together with the *Ph*-HOMO towards higher energies as a function of the film thickness [S1], so that the BE difference between the ring- (*R*-) and phenyl- (*Ph*-) related features is nearly constant, irrespective of the ZnTPP coverage.

Assuming that a similar relation holds also for the empty states, we infer the same Δ = 2.2 eV energy difference between the *R*-LUMO and *Ph*-LUMO states for both the 1 ML-and the 20 ML thick samples.

References

S1. Bussetti, G.; Calloni, A.; Celeri, M.; Yivlialin, R.; Bottegoni, F.; Finazzi, M.; Duò, L.; Ciccacci, F. *Appl. Surf. Sci.*, **2016**, *390*, 856-862.
